# Supplementary material for: Homodimer-mediated phosphorylation of C/EBPα-p42 S16 modulates acute myeloid leukaemia differentiation through liquid-liquid phase separation
Source: Nat Commun. 2023 Oct 30;14:6907. doi: 10.1038/s41467-023-42650-3 (PMC10616288; doi:10.1038/s41467-023-42650-3)
Supplement: Supplementary file 1 — Supplementary Information [file 41467_2023_42650_MOESM1_ESM.pdf]

Supplementry Figures

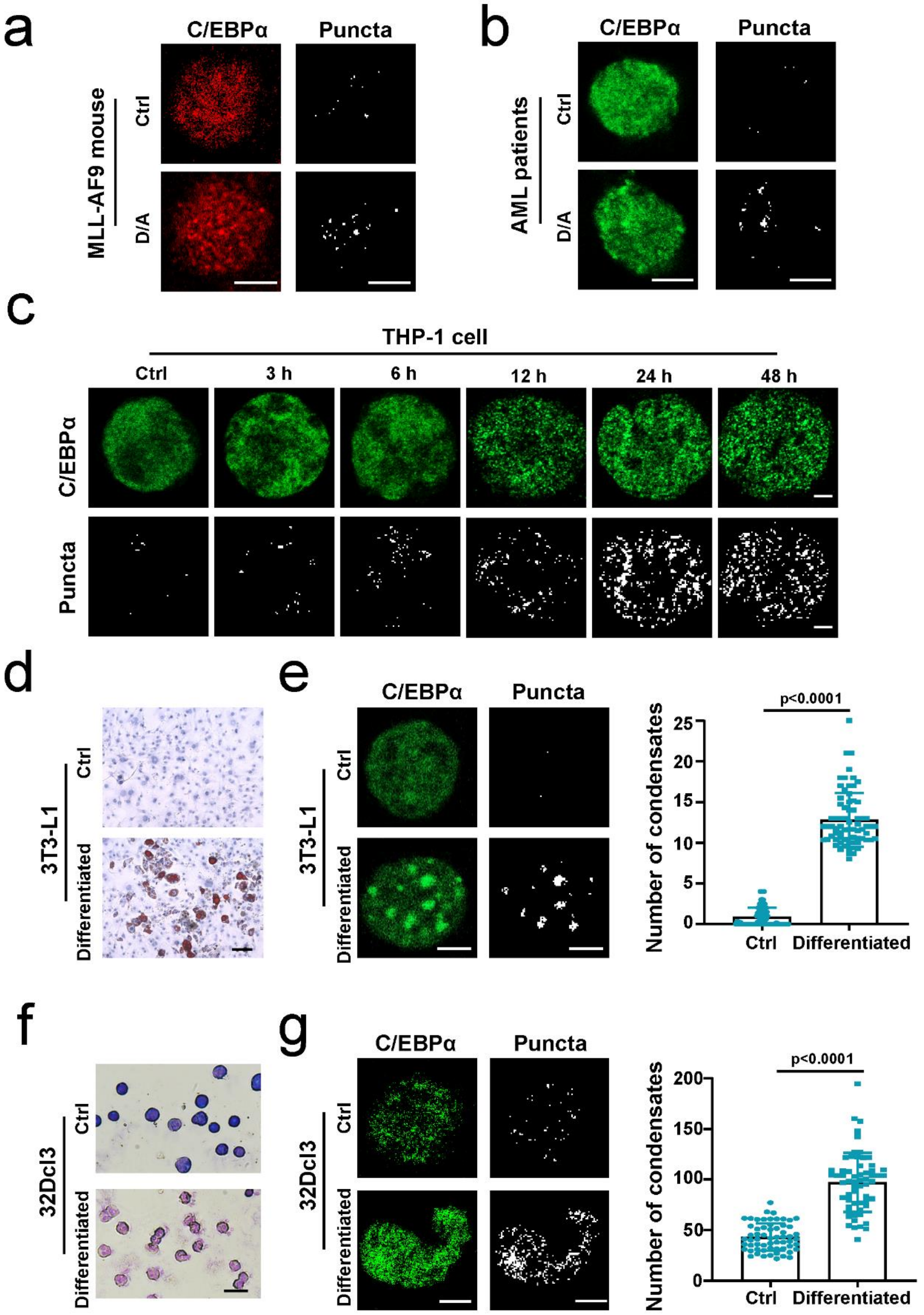

Supplementary Fig.1 | C/EBPα undergoes an abnormal particle alteration in different differentiation systems. a-c, The segmented puncta after quantification for Fig. 1d-f. d, Oil red O was used to confirm that 3T3-L1 cells differentiated into adipocytes (n = 3

biologically independent experiments). e, Immunofluorescence analysis of endogenous C/EBPα condensate in 3T3-L1 cells and adipocytes. Quantification of endogenous C/EBPα condensates in each cell (n = 3 biologically independent experiments). A two-tailed unpaired Student's *t*-test was used for statistical analysis, and data were presented as mean values ± SEM. f, Wright-Giemsa staining solution confirmed that 32Dcl3 cells differentiated into granulocytes (n = 3 biologically independent experiments). g, Immunofluorescence analysis of endogenous C/EBPα condensate in control and differentiated 32Dcl3. Quantification of endogenous C/EBPα condensates in each cell (n = 3 biologically independent experiments). A two-tailed unpaired Student's *t*-test was used for statistical analysis, and data were presented as mean values ± SEM. The experiments in e and g were observed at least 20 random fields in each experiment. For a, b, e and g, scar bars, 5 μm. For c, scar bar, 2 μm. For d and f, scar bar, 20 μm. Source data are provided as a Source Data file.

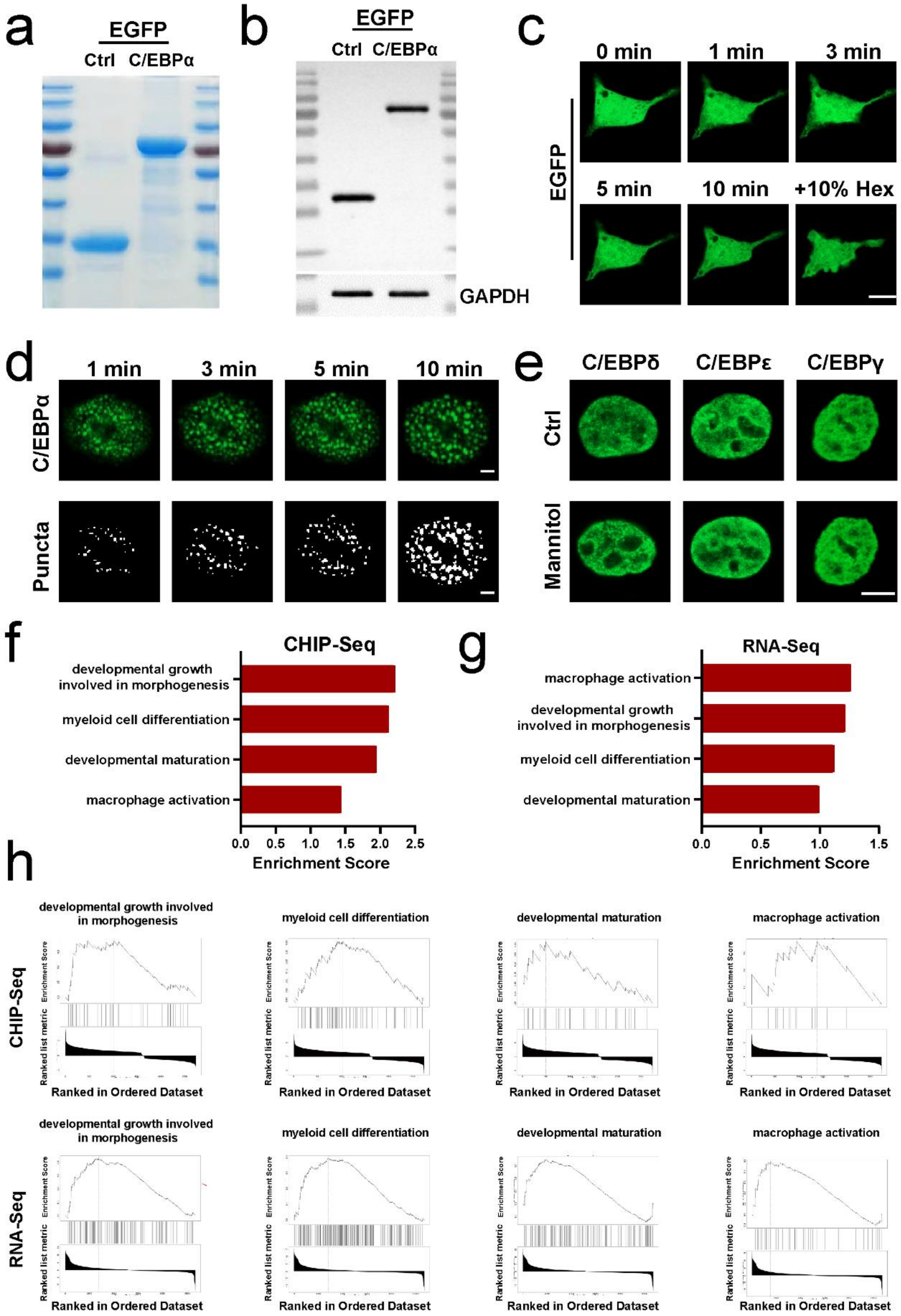

Supplementary Fig.2 | The LLPS of C/EBPα promoted its transcription ability. a, Coomassie blue staining of recombinant C/EBPα proteins purified from *E.coli*. b, HEK 293T cells transfected with EGFP and C/EBPα-EGFP were analysed using western blotting. c, Live-cell imaging of HEK 293T cells expressing EGFP at the indicated

times following mannitol treatment. d, The segmented puncta after quantification for Fig.2f. e, Live-cell imaging of HEK 293T cells expressing C/EBPδ, C/EBPε, C/EBPγ with or without mannitol (n = 3 biologically independent experiments). f and the upper line of h, the GSEA analysis of ChIP-seq showed that the phase-separated C/EBPα activated some differentiation-related pathways in PMA treated THP-1 cells. g and bottom line of the h, the GSEA analysis of RNA-seq showed the phase-separated of C/EBPα also activated the same differentiation-related pathways in PMA treated THP-1 cells (n = 3 biologically independent experiments). For c and e, scar bar, 5 μm. For d, scar bar, 2 μm. Source data are provided as a Source Data file.

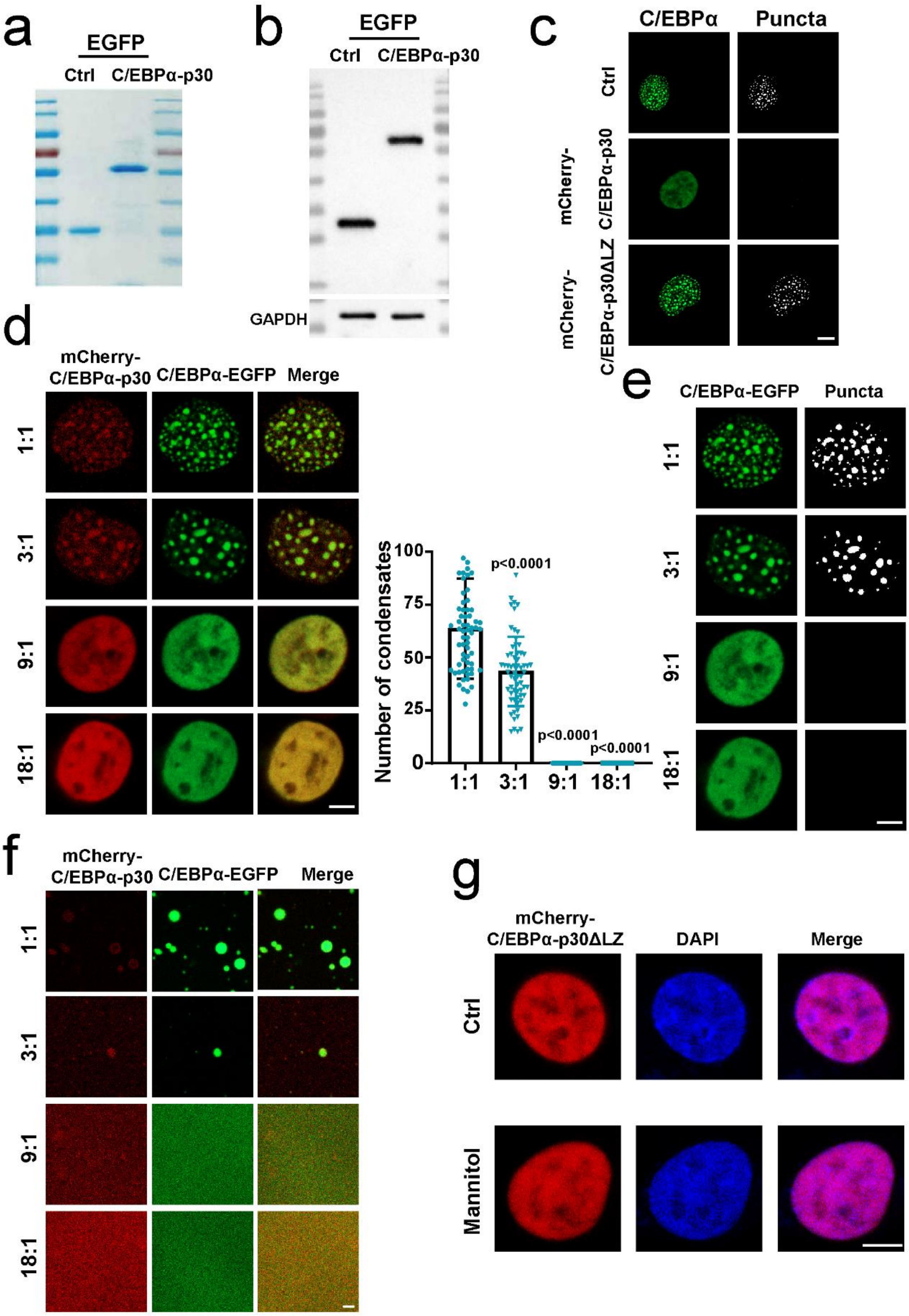

Supplementary Fig.3 | C/EBPα-p30 affects the LLPS C/EBPα and C/EBPα-p30 Δ LZ has no LLPS. a, Coomassie blue staining of recombinant C/EBPα-p30 proteins purified from *E.coli*. b, HEK 293T cells transfected with EGFP and C/EBPα-p30 were

analyzed using western blotting. c, The segmented puncta after quantification for Fig.3g. d, The images indicated that the number of C/EBPα-EGFP puncta in mannitol-treated HEK 293T cells. The mass ratio of C/EBPα-p30 construct to full-length C/EBPα construct were 1:1, 3:1, 9:1 and 18:1. The mass of C/EBPα-EGFP is 0.2 μg. Quantification of C/EBPα-EGFP puncta (n = 3 biologically independent experiments). A two-tailed unpaired Student's *t*-test was used for statistical analysis, and data were presented as mean values ± SEM. e, The segmented puncta after quantification for Supplementary Fig.3d. f, The images indicated the number of C/EBPα-EGFP puncta in the mixture of purified mCherry-tagged C/EBPα-p30 and C/EBPα-EGFP in the mass ratios of 1:1, 3:1, 9:1 and 18:1 (n = 3 biologically independent experiments). The mass of purified C/EBPα-EGFP protein is 14 ng. g, Images of HEK 293T cells that expressing mCherry-tagged C/EBPα-p30ΔLZ with or without mannitol. For c-e and g, scar bar, 5 μm. For f, scar bar, 2 μm. Source data are provided as a Source Data file.

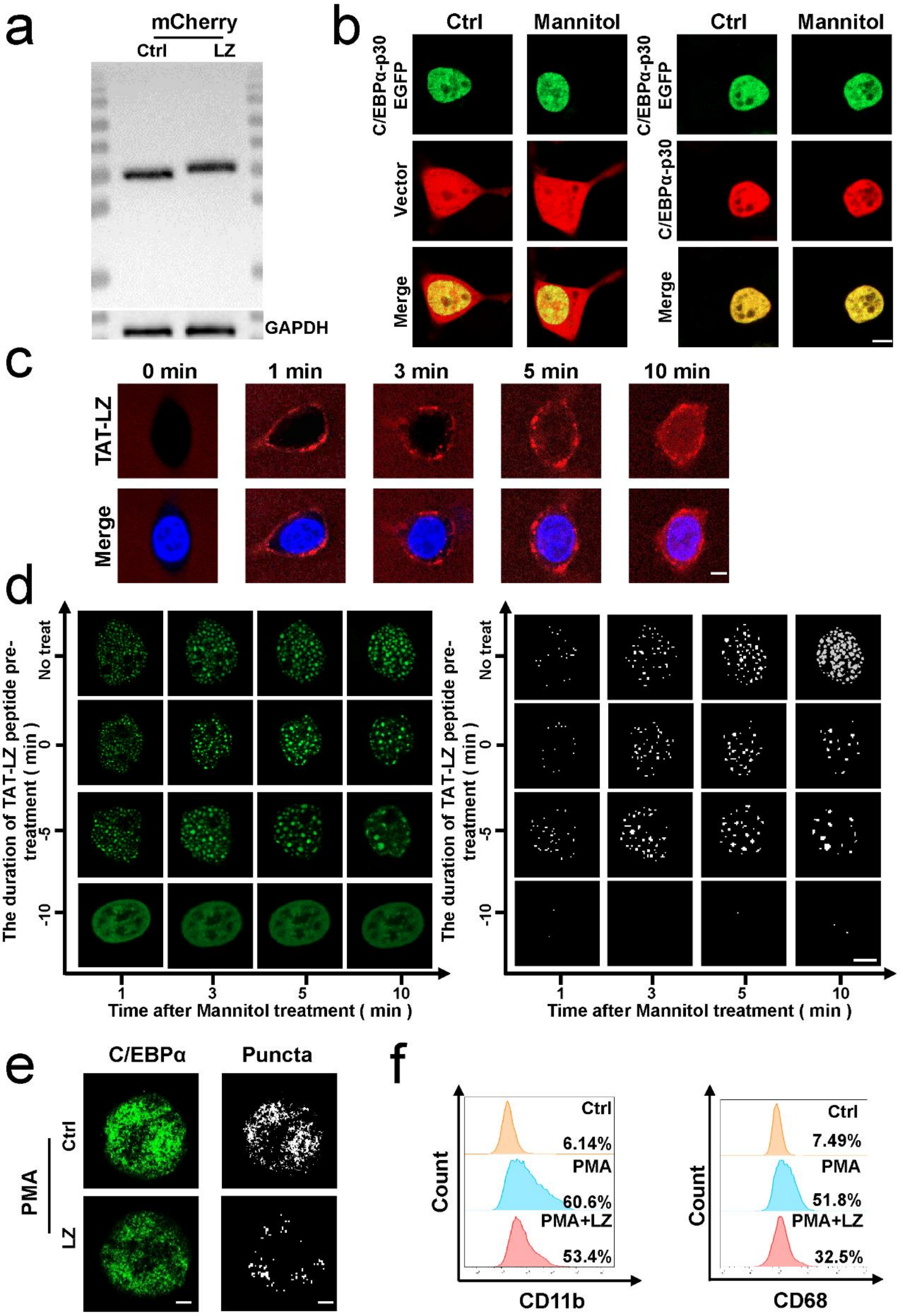

Supplementary Fig.4 | The LZ fragment inhibits the LLPS. a, Western blot analysis showed LZ fragment was constructed into HEK 293T cells. b, Live-cell images showed that the there is no phase separation in mannitol-treated HEK 293T cells transfected

with C/EBPα-p30-EGFP plasmid to mCherry-vector plasmids or C/EBPα-p30 at a molar ratio of 1:1 (n = 3 biologically independent experiments). c, Representative images showed the entry of 5 μM TAT-LZ into HEK 293T living cells at 0, 1 min, 3 min, 5 min and 10 min. d-e, The segmented puncta after quantification for Fig.4e and 4g. f, Representative flow analysis pictures for 4j. For b-d, scar bar, 5 μm. For e, scar bar, 2 μm. Source data are provided as a Source Data file.

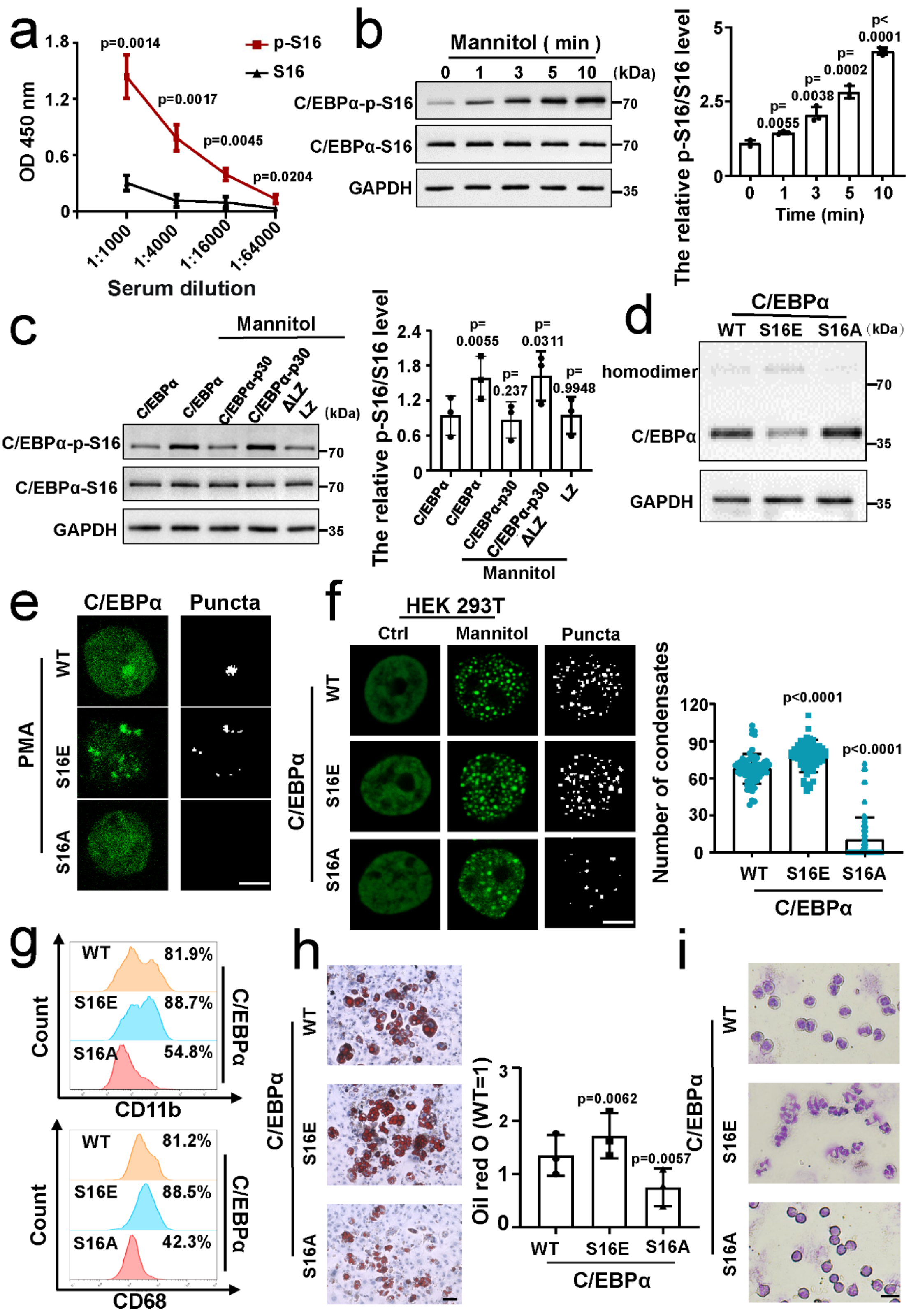

Supplementary Fig.5 | Phosphorylation of C/EBPα at S16 stabilizes its LLPS to promote differentiation. a, Elisa assay showed the specificity of anti-C/EBPα-p-S16 antibody (n = 3 biologically independent experiments). b, Western blot analysis showed

the elevated level of C/EBPα phosphorylation at S16 (p-S16) in HEK 293T cells transfected with C/EBPα-EGFP under mannitol treatment for 0, 1 min, 3 min, 5 min and 10 min. Relative p-S16 level (n = 3 biologically independent experiments). A two-tailed unpaired Student's *t*-test was used for statistical analysis, and data were presented as mean values ± SEM. c, Western blot analysis showed the level of C/EBPα p-S16 in HEK 293T cells transfected with the p30, p30-ΔLZ, or LZ together with C/EBPα-EGFP at a mass ratio of 9:1 after mannitol treatment. Relative p-S16 level (n = 3 biologically independent experiments). A two-tailed unpaired Student's *t*-test was used for statistical analysis, and data were presented as mean values ± SEM. d, The analysis of native gel electrophoresis showed that the changes of C/EBPα-p42 homodimers in the HEK 293T cells transfected with Control, C/EBPα p-S16E or C/EBPα p-S16A together with C/EBPα. e, The segmented puncta after quantification for Fig.5d. f, Representative images of HEK 293T living cells transfected with C/EBPα-S16E-EGFP, C/EBPα-EGFP (WT) or C/EBPα-S16A-EGFP without or with mannitol and the segmented puncta after quantification. Quantification of C/EBPα condensates. A two-tailed unpaired Student's *t*-test was used for statistical analysis, and data were presented as mean values ± SEM. g, Representative flow analysis pictures for 5g. h, Oil red O assay showed the effect of C/EBPα-S16 on differentiation of 3T3-L1 into adipocytes. Quantification of Oil red O staining (n = 3 biologically independent experiments). A two-tailed unpaired Student's *t*-test was used for statistical analysis, and data were presented as mean values ± SEM. i, Wright-Giemsa staining showed the effect of C/EBPα-S16 on differentiation of 32Dcl3 cells into granulocytes (n = 3 biologically independent experiments). For a-c, f and h, a two-tailed unpaired Student's *t*-test was used for statistical analysis, and data were presented as mean values ± SEM. For e and f, scar bar, 5 μm. For h and i, scar bar, 20 μm. Source data are provided as a Source

Data file.

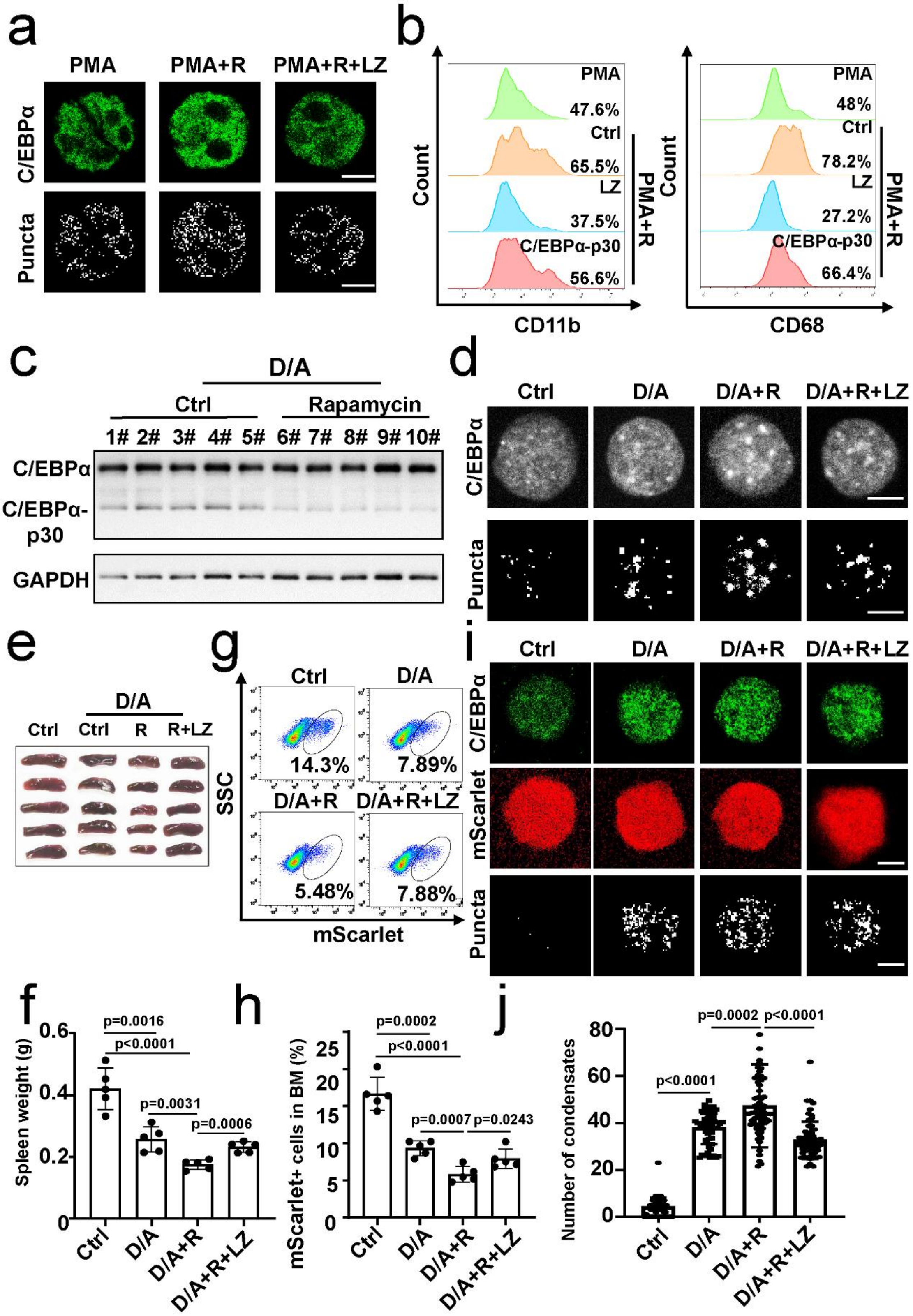

Supplementary Fig.6 | Decreasing the endogenous C/EBPα-p30/C/EBPα ratio rescues the function of C/EBPα through its LLPS. a, The segmented puncta after

quantification for Fig.6b. b, Representative flow analysis pictures for Fig. 6d. c, Western blot analysis showed that rapamycin affect ratio of C/EBPα-p30/C/EBPα-p42 in Ara-C and Dox-treated MLL-AF9 mice (n = 5 per group). d, The segmented puncta after quantification for Fig.6h. e-f, In the AML xenograft model, on the 40th day, the representative photographs and weights of spleens isolated from mScarlet+ GFP+ MLL-AF9 mice in the four groups (n = 5 per group). g-h, In the AML xenograft model, flow cytometric analysis of mScarlet+ leukaemia cells in BM of the four groups and quantification of mScarlet+ leukaemia cells (n = 5 per group). i, In the AML xenograft model, immunofluorescence photograph analysis of the number of endogenous C/EBPα condensates in the control, D/A, D/A+R and D/A+R+LZ groups (n = 5 per group). j, quantification of endogenous C/EBPα condensates in each cell. For f-j, a two-tailed unpaired Student's *t*-test was used for statistical analysis, and data were presented as mean values ± SEM. For a, d, i, scar bar, 5 μm. Source data are provided as a Source Data file.
