# Supplementary material for: Homodimer-mediated phosphorylation of C/EBPα-p42 S16 modulates acute myeloid leukaemia differentiation through liquid-liquid phase separation
Source: Nat Commun. 2023 Oct 30;14:6907. doi: 10.1038/s41467-023-42650-3 (PMC10616288; doi:10.1038/s41467-023-42650-3)
Supplement: Supplementary file 3 — Reporting Summary [file 41467_2023_42650_MOESM3_ESM.pdf]

nature portfolio

Corresponding author(s): Chunyan Ji

Last updated by author(s): Sep 1, 2023

Reporting Summary

Nature Portfolio wishes to improve the reproducibility of the work that we publish. This form provides structure for consistency and transparency in reporting. For further information on Nature Portfolio policies, see our Editorial Policies and the Editorial Policy Checklist.

Statistics

For all statistical analyses, confirm that the following items are present in the figure legend, table legend, main text, or Methods section.

n/a	Confirmed	
☐	☒	The exact sample size (*n*) for each experimental group/condition, given as a discrete number and unit of measurement
☐	☒	A statement on whether measurements were taken from distinct samples or whether the same sample was measured repeatedly
☐	☒	The statistical test(s) used AND whether they are one- or two-sided<br>*Only common tests should be described solely by name; describe more complex techniques in the Methods section.*
☒	☐	A description of all covariates tested
☒	☐	A description of any assumptions or corrections, such as tests of normality and adjustment for multiple comparisons
☐	☒	A full description of the statistical parameters including central tendency (e.g. means) or other basic estimates (e.g. regression coefficient) AND variation (e.g. standard deviation) or associated estimates of uncertainty (e.g. confidence intervals)
☐	☒	For null hypothesis testing, the test statistic (e.g. *F*, *t*, *r*) with confidence intervals, effect sizes, degrees of freedom and *P* value noted<br>*Give P values as exact values whenever suitable.*
☒	☐	For Bayesian analysis, information on the choice of priors and Markov chain Monte Carlo settings
☒	☐	For hierarchical and complex designs, identification of the appropriate level for tests and full reporting of outcomes
☐	☒	Estimates of effect sizes (e.g. Cohen's *d*, Pearson's *r*), indicating how they were calculated

Our web collection on statistics for biologists contains articles on many of the points above.

Software and code

Policy information about availability of computer code

Data collection	No software were used
Data analysis	GraphPad Prism 9 and Image J.

For manuscripts utilizing custom algorithms or software that are central to the research but not yet described in published literature, software must be made available to editors and reviewers. We strongly encourage code deposition in a community repository (e.g. GitHub). See the Nature Portfolio guidelines for submitting code & software for further information.

Data

Policy information about availability of data

All manuscripts must include a data availability statement. This statement should provide the following information, where applicable:

- Accession codes, unique identifiers, or web links for publicly available datasets
- A description of any restrictions on data availability
- For clinical datasets or third party data, please ensure that the statement adheres to our policy

The raw sequencing data of CHIP-Seq and RNA-Seq generated from this study have been deposited to the GEO database under the accession codes GSE243422, GSE243423 and GSE243424.

Research involving human participants, their data, or biological material

Policy information about studies with human participants or human data. See also policy information about sex, gender (identity/presentation), and sexual orientation and race, ethnicity and racism.

Reporting on sex and gender	We obtained AML primary cell from from 3 male and 3 female patients.
Reporting on race, ethnicity, or other socially relevant groupings	They are from China, Han group.
Population characteristics	The newly diagnosed AML with normal karyotype without any treatment was collected. The age were 23-48 years old with FLT3, NPM1 ,Kras mutant or wild type.
Recruitment	People voluntarily participated in the study by signing informed consent form. Although there are differences in age or gene mutation, it has not affected the conclusion in this study.
Ethics oversight	The study was approved by the Human Ethics Committee of the Shandong University (SDULCLL2020-1-14).

Note that full information on the approval of the study protocol must also be provided in the manuscript.

Field-specific reporting

Please select the one below that is the best fit for your research. If you are not sure, read the appropriate sections before making your selection.

☒ Life sciences ☐ Behavioural & social sciences ☐ Ecological, evolutionary & environmental sciences

For a reference copy of the document with all sections, see nature.com/documents/nr-reporting-summary-flat.pdf

Life sciences study design

All studies must disclose on these points even when the disclosure is negative.

Sample size	Sample sizes were chosen based on preliminary data demonstrating statistically significant differences for each specific assay.
Data exclusions	The exclusion criteria were as follows: age <15 years and > 70 years, diarrhea, receiving antibiotics or hormone therapy within the last 10 weeks, and blood pressure anomalies.
Replication	All experiments were performed with at least three technical replicates on more than one occasion to ensure reproducibility across experiments.
Randomization	The mice used in the experiments were randomly divided into different treatment groups. We also randomly selected one subset from the participants meeting the inclusion criteria.
Blinding	Blinding was not relevant as all processing methods were done through available software with consistent parameters utilized across all treatment groups.

Reporting for specific materials, systems and methods

We require information from authors about some types of materials, experimental systems and methods used in many studies. Here, indicate whether each material, system or method listed is relevant to your study. If you are not sure if a list item applies to your research, read the appropriate section before selecting a response.

Materials & experimental systems

n/a	Involved in the study	
☐	☒	Antibodies
☐	☒	Eukaryotic cell lines
☒	☐	Palaeontology and archaeology
☐	☒	Animals and other organisms
☒	☐	Clinical data
☒	☐	Dual use research of concern
☒	☐	Plants

Methods

n/a	Involved in the study	
☐	☒	ChIP-seq
☐	☒	Flow cytometry
☒	☐	MRI-based neuroimaging

Antibodies

Antibodies used	The following antibodies and reagents were purchased from the indicated companies: anti-C/EBPα (Cell Signaling Technology, 2295;for IF, 1:50; for WB,1:1000); anti-mCherry (Invitrogen, M11217;1:1000); anti-EGFP (Santa Cruz, sc-9996;1:2000); anti-CD11b (101226, BioLegend;1:20); anti-CD68 (333805, BioLegend;1:20); anti-Flag (Proteintech, 66008-4-Ig;1:1000); anti-GAPDH (Abways, AB0038;1:5000); anti-C/EBPα(Cell Signaling Technology, #8178; For Chip-Seq, 1:100); Goat Anti-Mouse IgG antibody (Abways, AB0102, 1:5000)
Validation	All the antibodies used in this study are commercially available and have been verified by the manufacturers according to the data on their websites

Eukaryotic cell lines

Policy information about cell lines and Sex and Gender in Research

Cell line source(s)	The primary cells from AML patients; HEK 293T and THP-1 were purchased from the Institute of Hematology and Blood Diseases Hospital, Chinese Academy of Medical Sciences and Peking Union Medical College, Tianjin, China; 32Dcl3 were sourced from Meisen CTCC, Zhejiang, China. 3T3-L1 were purchased from National collection of Authenticated Cell Cultures.
Authentication	For 293T , 3T3-L1 and THP-1 were authenticated by STR profiling. For 32DCL3 were authenticated by PCR assays.
Mycoplasma contamination	Mycoplasma was detected by Hoechst 33258, and all cell lines have no mycoplasma.
Commonly misidentified lines (See ICLAC register)	No

Animals and other research organisms

Policy information about studies involving animals; ARRIVE guidelines recommended for reporting animal research, and Sex and Gender in Research

Laboratory animals	C57/B6 male mice aged 6-8 weeks and male NOD/SCID mice aged 3 to 4 weeks were purchased from Charles River and were maintained in the laboratory animal centre of Clinical Medical College, Shandong University. Mice were maintained on a 12-h dark/light cycle at ambient temperature (72±2F) with controlled humidity (~45%).
Wild animals	The study did not involve wild animals.
Reporting on sex	All mice were male.
Field-collected samples	The study did not involve field-collected samples.
Ethics oversight	Animal protocols were approved by the Animal Ethics Committee of Shandong University (SDULCLL2020-2-12).

Note that full information on the approval of the study protocol must also be provided in the manuscript.

Plants

Seed stocks	*Report on the source of all seed stocks or other plant material used. If applicable, state the seed stock centre and catalogue number. If plant specimens were collected from the field, describe the collection location, date and sampling procedures.*
Novel plant genotypes	*Describe the methods by which all novel plant genotypes were produced. This includes those generated by transgenic approaches, gene editing, chemical/radiation-based mutagenesis and hybridization. For transgenic lines, describe the transformation method, the number of independent lines analyzed and the generation upon which experiments were performed. For gene-edited lines, describe the editor used, the endogenous sequence targeted for editing, the targeting guide RNA sequence (if applicable) and how the editor was applied.*
Authentication	*Describe any authentication procedures for each seed stock used or novel genotype generated. Describe any experiments used to assess the effect of a mutation and, where applicable, how potential secondary effects (e.g. second site T-DNA insertions, mosiacism, off-target gene editing) were examined.*

ChIP-seq

Data deposition

☒ Confirm that both raw and final processed data have been deposited in a public database such as GEO.

☒ Confirm that you have deposited or provided access to graph files (e.g. BED files) for the called peaks.

Data access links<br>*May remain private before publication.*	https://figshare.com/s/1112b2e6202990bf2dfa
Files in database submission	H0169
Genome browser session<br>(e.g. UCSC)	https://figshare.com/s/1112b2e6202990bf2dfa

Methodology

Replicates	We used a set of THP-1 and THP-1 treated with PMA for CHIP-seq.
Sequencing depth	Raw reads were filtered to obtain high-quality clean reads by removing sequencing adapters, short reads (length <35bp) and low-quality reads using trim-galore (v0.6.4). Then FastQC (v0.11.9) and Multiqc (v1.8) is used to ensure high reads quality.
Antibodies	C/EBPα, Cell Signaling Technology, #8178; Drosophila H2A.v, Active Motif, 61686;
Peak calling parameters	Peak detection was performed using the MACS2 (v2.2.6) peak finding algorithm with 0.05 set as the q-value cutoff.
Data quality	1×107 THP-1 or PMA-treated THP-1 cells were fixed in 1% formaldehyde at room temperature for 10 minutes, quenched with 125 mM glycine and harvested. The harvested cells were sent to Active Motif China (Shanghai, China) for ChIP-Seq. Active Motif prepared chromatin, performed ChIP reactions, generated the libraries and performed basic data analysis. In brief, chromatin was isolated by adding lysis buffer and fragmented by sonication. DNA was sheared to an average length of 200-500 bp with EpiShear probe sonicator (Active Motif, 53051). The same amount of Drosophila chromatin (Active Motif, 53083) was incubated with sheared chromatins from THP-1 cells and PMA-treated PMA cells, respectively for ChIP normalization. Genomic DNA (Input) was prepared by de-crosslinking at 65°C for 4 hours followed by RNase A and Proteinase K digestion. The Input DNA was purified with PCR Purification Kit (Qiagen, 28004) and quantified (Onedrop, Wins). Fragmented chromatin mix (for IP) was incubated with 5 µl of anti-C/EBPα (Cell Signaling Technology, 8178) and 1 µg of Drosophila H2A.v antibody (Active Motif, 61686) at 4°C for overnight. 25 µl of rProtein G Magarose Beads (Smart-Lifesciences, SM004005) were added to the samples and incubated at 4°C for 2 hours. Complexes were washed, eluted from the beads with SDS buffer, then de-crosslinked at 65°C for overnight. The de-crosslinked chromatin was subjected to RNase A and proteinase K treatment. ChIP DNA was purified with PCR Purification Kit (Qiagen, 28004) and quantified (Thermo Fisher, Qubit).
Software	Raw reads were filtered to obtain high-quality clean reads by removing sequencing adapters, short reads (length <35bp) and low-quality reads using trim-galore (v0.6.4). Then FastQC (v0.11.9) and Multiqc (v1.8) is used to ensure high reads quality. The clean reads were mapped to the human genome (assembly human genome hg38, primary genome) using the Burrow-Wheeler Aligner (BWA v0.7.17) software. The clean reads were mapped to the Drosophila genome (assembly Drosophila genome dm6, spike-in genome) using the Burrow-Wheeler Aligner (BWA v0.7.17) software. PCR duplicates were removed using Picard (v2.22.2-0). Scale factors were calculated by formula (number of the clean reads mapped to spike-in genome/the maximum of numbers of the clean reads mapped to spike-in genome). Subset of the clean reads were determined by randomly downsampling using bamtools (V2.5.1), the numbers of reads to keep were calculated by formula (number of the clean reads mapped to primary genome). Peak detection was performed using the MACS2 (v2.2.6) peak finding algorithm with 0.05 set as the q-value cutoff. Annotation of peak sites to gene features was performed using the homer annotatePeaks.pl (v4.10). Pearson correlation coefficient between biological replicates was calculated using deeptools (v3.4.3) with default parameters. The resulting histograms were stored in bigWig files using deeptools (v3.4.3).

Flow Cytometry

Plots

Confirm that:

☒ The axis labels state the marker and fluorochrome used (e.g. CD4-FITC).

☒ The axis scales are clearly visible. Include numbers along axes only for bottom left plot of group (a 'group' is an analysis of identical markers).

☒ All plots are contour plots with outliers or pseudocolor plots.

☒ A numerical value for number of cells or percentage (with statistics) is provided.

Methodology

Sample preparation	For all flow cytometry assays, following treatment or post-treatment incubation, cells were washed 2x in PBS.5×10^5 cells were resuspended in 100 ul of pbss, and antibody was incubated for 30 minutes in the dark.
Instrument	Beckman Counter
Software	FlowJo 10.0.7
Cell population abundance	Cells were run to achieve > =20,000 events in the gated cell population

Gating strategy	Gating was performed based on identifying a distinct population in FSC vs SSC plots.

☒ Tick this box to confirm that a figure exemplifying the gating strategy is provided in the Supplementary Information.
